# Arthroereisis with a Talar Screw in Symptomatic Flexible Flatfoot in Children

**DOI:** 10.3390/jcm12237475

**Published:** 2023-12-02

**Authors:** Andrzej Bobiński, Łukasz Tomczyk, Marcin Pelc, Damian Aleksander Chruścicki, Bartosz Śnietka, Piotr Morasiewicz

**Affiliations:** 1 Department of Orthopaedic and Trauma Surgery, Institute of Medical Sciences, University of Opole, Witosa 26, 45-401 Opole, Poland; bobinski@interia.eu; 2 Department of Food Safety and Quality Management, Poznan University of Life Sciences, Wojska Polskiego 28, 60-637 Poznan, Poland; 3 Institute of Medical Sciences, University of Opole, Witosa 26, 45-401 Opole, Poland

**Keywords:** sport, function, pain, pes planovalgus, subtalar arthroereisis, Spherus screw

## Abstract

Background: Pes planovalgus, or flexible flatfoot, deformity is a common problem in pediatric orthopedic patients. There is no consensus on using the technique of arthroereisis in the treatment of symptomatic pes planovalgus. The aim of our study was to prospectively assess the functional outcomes following symptomatic pes planovalgus treatment with the use of the Spherus talar screw. Methods: Twenty-seven patients (11 females, 16 males), at a mean age of 10.5 years (7–14 years) were included in the prospective study. We assessed the level of physical activity (including sports) based on the University of California, Los Angeles (UCLA) activity scale, a 10-point level-of-activity VAS scale, and the Grimby physical activity scale. Pain was assessed based on a VAS pain scale; foot function was assessed with the revised Foot Function Index (FFI-R); and ankle joint mobility was measured. Results: The mean follow-up period was 18 months (14–26 months). There was a significant improvement in VAS-measured physical activity scores from 5.47 to 7 at follow-up, *p* = 0.048. There was a significant improvement in UCLA activity scale scores from 4.78 to 6.05 at follow-up, *p* = 0.045. Pain levels decreased from a mean VAS score of 4.73 prior to surgery to a mean score of 2.73 at follow-up, *p* = 0.047. The functional FFI-R scores showed a significant improvement from 140 points prior to surgery to 97.75 points at follow-up, *p* = 0.017. Comparison of the preoperative and follow-up values of the range of plantar flexion, adduction, and abduction in the operated limb also showed no significant changes in those individual parameters. The mean values of dorsiflexion, plantar flexion, adduction, and abduction at the ankle joint at follow-up, compared individually between the operated and non-operated foot showed no statistically significant differences. Conclusions: The use of a talar screw in the treatment of symptomatic pes planovalgus helps reduce pain and improve functional outcomes after treatment. Foot function assessments showed diminished pain, improved levels of physical and sport activity, and no effect on the range of motion after surgery in comparison with preoperative data. Arthroereisis with a talar screw is a valid surgical technique for the treatment of symptomatic pes planovalgus.

## 1. Introduction

Pes planovalgus, or flexible flatfoot, deformity is a common problem in pediatric orthopedic patients [1,2,3,4,5,6,7,8,9,10,11,12,13,14,15,16,17,18,19,20,21,22]. Asymptomatic cases are not an indication for treatment [1,2,5,6,7,8,10,12,13,16,18,19,20,22]. However, the deformity may cause foot pain, limping, asymmetric gait, pain in the ankle joint and leg, limitations in daily activities, gait inefficiency, and limited sport activity [1,2,3,4,5,6,7,8,12,13,16,20,22]. The lack of treatment of symptomatic pes planovalgus in pediatric patients may lead to the development of various musculoskeletal conditions in adulthood; these conditions include hallux valgus deformity, Morton’s neuroma, and degenerative lesions in the foot and ankle joint [2,23]. The initial approach to symptomatic pes planovalgus is conservative treatment continued for 3–6 months, which involves walking restrictions, rehabilitation, shoe inserts, body weight reduction, and the use of analgesics [1,2,5,6,7,8,12,13,18,19,22]. If the conservative approach is ineffective, surgical treatment is recommended [1,2,4,5,6,7,8,12,13,18,19,20,22]. 

There are many known techniques for surgical treatment used in cases of symptomatic pes planovalgus [1,2,3,4,5,6,7,8,9,10,11,12,13,14,15,16,17,18,19,20,21,22]. These include arthroereisis, calcaneal or talar osteotomy, arthrodesis (of the calcaneocuboid, subtalar, and talonavicular joints), soft-tissue surgery, Achilles tendon lengthening, gastrocnemius lengthening, tendon transfer, and arthroereisis [1,2,3,4,5,6,7,8,9,10,11,12,13,14,15,16,17,18,19,20,21,22]. Arthroereisis is a minimally invasive procedure involving the temporary blocking of the subtalar joint through sinus tarsi implant placement, calcaneal screw insertion, or a small wedge-shaped bone graft implantation between the talus and calcaneus [1,2,3,4,5,6,7,8,9,10,11,12,13,14,15,16,17,18,19,20,21,22]. The purpose of arthroereisis is to limit foot pronation and forefoot abduction, provide lateral support for the subtalar joint, and reduce hindfoot eversion [1,2,3,5,13,22]. Implant placement helps increase both foot supination and forefoot adduction [3,13]. Implants used for arthroereisis procedures also affect proprioception due to their mechanical effect on the numerous proprioceptors found in the sinus tarsi [1,2,3,13,22]. The best surgical outcomes have been reported for arthroereisis procedures conducted in patients aged 7–14 years; this is because the implants affect the remodeling of the still growing talus and calcaneus and the three-dimensional structure of the subtalar joint [2,4,5,6,8,10,12,13,21]. 

Since it is a minimally invasive, inexpensive, and time-efficient procedure with few complications, arthroereisis is an accepted method of treatment for symptomatic pes planovalgus, yielding high rates of patient satisfaction with treatment [1,2,3,5,6,7,8,9,19,22]. Nonetheless, there is no consensus on the technique of arthroereisis in the treatment of symptomatic pes planovalgus [1,2,4,5,7,8,10,12,13,20]. Some authors suggest the use of free-floating sinus tarsi implants [6,7,9], some advocate for inserting screws into the calcaneus [3,8,13,15,16,19], still others report comparable outcomes irrespective of the type of implant used [1,2,12,18]. The goals of arthroereisis procedure includes reduction of pain and deformity, improvement of gait, and increase in physical activity and sport activity [5,7,8,10,12].

In our previous study we assessed short-term and medium-term radiological and clinical arthroereisis outcomes achieved with a talar Spherus screw, in the same group of patients included in the current study [22]. We reported radiological improvement and good clinical outcomes [22]. However, there is a paucity of studies assessing physical and sport activity following arthroereisis [5].

To the best of our knowledge, there have been no studies assessing foot function, range of motion in the ankle joint, pain severity, and patients’ physical and sport activity following talar screw insertion as part of arthroereisis treatment of symptomatic pes planovalgus. Some studies on arthroereisis are retrospective in nature [1,3,4,5,6,7,12,13,14,15,19]; therefore, we undertook a prospective assessment. 

We hypothesized that arthroereisis with the use of a talar implant would improve functional outcomes.

The aim of our study was to prospectively assess the functional outcomes following symptomatic pes planovalgus treatment with the use of a Spherus talar screw. 

## 2. Materials and Methods

This was a prospective study conducted at a single center. We analyzed every patient with pes planovalgus who underwent arthroereisis with a Spherus talar screw in the period between 2021 and 2022. All patients underwent physical and radiological examinations and were diagnosed with symptomatic pes planovalgus based on the following signs and symptoms: foot pain, flexible flatfoot deformity, a talocalcaneal angle (kite angle) of >40 degrees measured on a weight-bearing foot radiograph [1,2,3,5,19]. All analyzed patients had undergone initial conservative treatment in the form of rehabilitation, shoe inserts, walking restrictions, non-steroidal anti-inflammatory drugs, and weight loss reduction over a period of 3–6 months [1,5,7,8,19]. 

**Figure 1:**
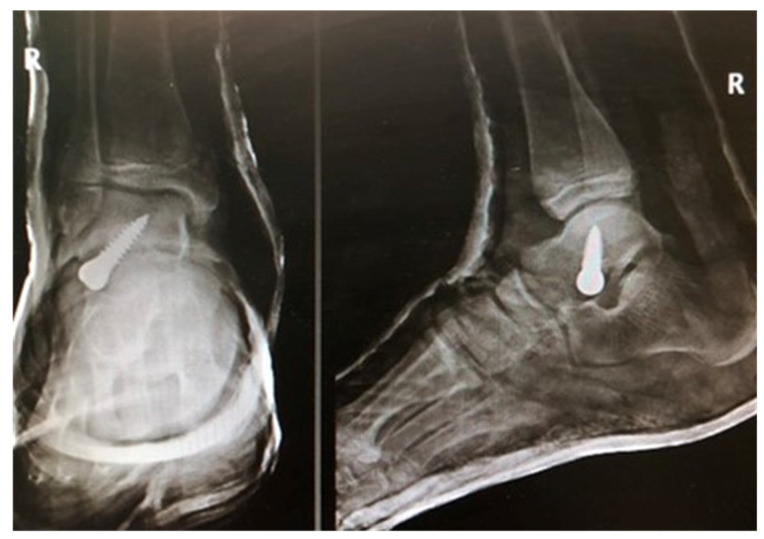
Radiographic images after pes planovalgus correction with a Spherus screw.

The study inclusion criteria were age between 7 and 14 years, symptomatic flexible flatfoot, failure of conservative treatment, arthroereisis with a Spherus talar screw (Gruppo Bioimpianti S.R.L., Milan, Italy), Figure 1, a follow-up period of at least 14 months, informed consent, available and complete medical and radiological records, available and complete functional assessments records (activity questionnaires, pain visual analog scale [VAS] scores, ankle range of motion), no history of lower limb injury, the absence of synostoses in radiographic images, and no neurological comorbidities. 

The exclusion criteria were ages under 7 years and over 14 years, history of lower limb injury, radiographic evidence of synostosis in the foot or the ankle joint, neurological comorbidities, a follow-up period of less than 14 months, incomplete medical, radiographic, or functional assessment records. All patients and their legal guardians were informed of the voluntary nature of their participation. The study, which was conducted in accordance with the Declaration of Helsinki, had been approved by an ethics committee.

In the period from the year 2021 to 2022, there were 35 arthroereisis procedures with the use of a Spherus talar screw conducted at our clinic. Application of the inclusion and exclusion criteria yielded 27 patients (11 females, 16 males), at a mean age of 10.5 years (7–14 years) who were ultimately included in the study. 

Patients with foot dorsiflexion limited to 5–10 degrees [13,14,18] underwent simultaneous Achilles tendon lengthening (Z-plasty). There were 10 such patients (37%). Each surgery was performed by one of two experienced orthopedic surgeons. The patients were operated on in a supine position, under tourniquet-induced ischemia of the treated lower limb. A small, 1–2-cm-long, incision was performed distal and slightly anterior to the tip of the lateral malleolus, and close to the sinus tarsi. Anteroposterior (AP) and lateral radiographs were performed intraoperatively to determine the appropriate location for implant insertion. Depending on patient’s age, body weight, foot size, and prior experience of the operator, the diameter of the selected screw was either 6.5 mm or 8 mm, and screw length ranged from 25 mm to 40 mm. With the patient’s foot in maximum supination, the Spherus screw was inserted into the inferior surface of the talus from the direction of the sinus tarsi, while being monitored via intraoperative AP and lateral radiography. On an AP view, the course of the introduced Spherus screw was oblique and directed superomedially, whereas, in a lateral view, the image of the screw overlapped with that of the lateral malleolus. Immediately after surgery, the patients were allowed to walk while bearing full weight on the operated foot; however, in case of pain, the use of two elbow crutches was recommended for up to 14 days following surgery. Six weeks after surgery, the patients were allowed to resume physical activity fully, including sports. The patients were followed-up radiographically at an orthopedic outpatient clinic six weeks after surgery and every three months thereafter.

As part of the study, we assessed the level of physical activity (including sports) based on the 6-point University of California, Los Angeles (UCLA) activity scale [24], a 10-point level-of-activity VAS scale [25], and a 6-point Grimby physical activity scale [26]. Pain was assessed based on a 10-point VAS pain scale; foot function was assessed with the revised Foot Function Index (FFI-R) [27]; and ankle joint mobility was measured manually with a goniometer. Dorsiflexion, plantar flexion, adduction, and abduction at the ankle joint were assessed both in the operated and non-operated limb, with the results expressed in degrees. These assessments were conducted both prior to surgery and at a long-term follow-up visit. 

### Statistical Analysis

Data were statistically analyzed using Statistica 13.1. The Shapiro–Wilk test was used to check for normality of distribution. The Mann–Whitney U test was used to compare quantitative variables. The level of statistical significance was set at *p* < 0.05.

## 3. Results

The mean follow-up period was 18 months (14–26 months). Detailed results of the physical activity, functional, and range-of-motion assessments have been presented in Table 1 and Table 2. We observed improvements in the UCLA activity scale scores from a mean score of 4.78 prior to surgery to a mean score of 6.05 at follow-up, *p* = 0.045; see Table 1.

Grimby activity scores showed an increase in physical activity from 3.26 prior to treatment to 3.94 at long-term follow-up; however, the difference was not statistically significant; see Table 1. There was a significant improvement in VAS-measured physical activity scores from a mean preoperative score of 5.47 to 7 at follow-up, *p* = 0.048, Table 1. Pain levels also showed improvement, decreasing from a mean VAS score of 4.73 prior to surgery to a mean score of 2.73 at follow-up; the difference was statistically significant, *p* = 0.047; see Table 1. 

**Figure 2:**
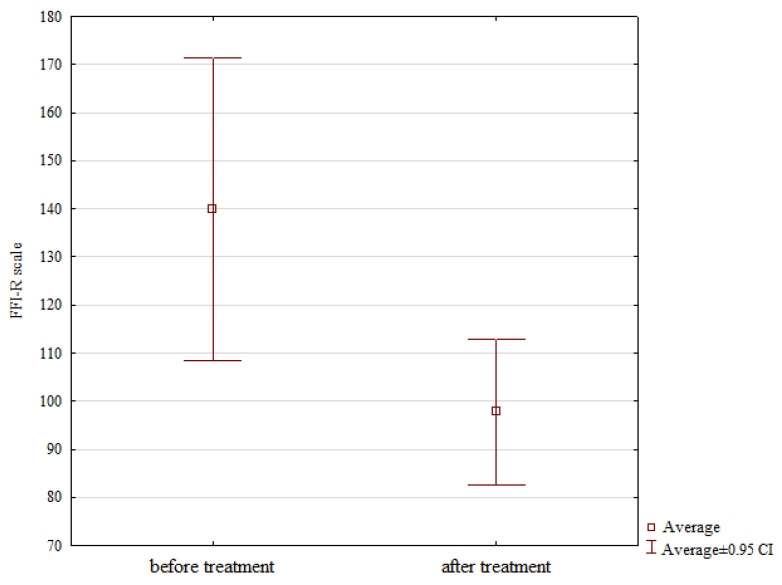
Preoperative and postoperative scores FFI-R scale scores values.

The functional scores assessed with the FFI-R scale also showed significant improvement from a mean score of 140 points prior to surgery to 97.75 points at follow-up, *p* = 0.017; see Table 1, Figure 2.

Dorsiflexion increased, though non-significantly, from a mean of 22° prior to surgery to a mean of 25.33° at follow-up; see Table 1. Comparison of the preoperative and follow-up values of the range of plantar flexion, adduction, and abduction in the operated limb also showed no significant changes in those individual parameters; see Table 1. 

Importantly, the mean values of dorsiflexion, plantar flexion, adduction, and abduction at the ankle joint at follow-up, compared individually between the operated and non-operated foot, showed no statistically significant differences; see Table 2. 

## 4. Discussion

The aim of our study was to assess whether the use of a talar screw for arthroereisis would improve functional parameters of the foot in symptomatic pes planovalgus. We observed improvement in the UCLA and VAS physical activity scores following treatment. There was also improvement in the FFI-R scores at follow-up and the VAS showed pain reduction following treatment, with no postoperative decrease in the range of motion at the ankle joint. The obtained results support our initial hypothesis. 

Arthroereisis, which is used in the treatment of symptomatic pes planovalgus, is minimally invasive, inexpensive, produces few complications, and ensures high rates of patient satisfaction [1,2,4,5,6,7,8,9,15,17,19]. The implants used for arthroereisis mechanically limit foot pronation and improve proprioception, which helps achieve the correction of pes planovalgus deformity [1,2,3,5]. The effects on proprioception induce changes in the interactions between muscles and ligaments [1,2,3,12,13].

To date, there has been no consensus among orthopedic surgeons regarding which type of arthroereisis implants to choose in patients with symptomatic pes planovalgus [1,2,5,7,8]. In our previous study, we assessed the short-term and medium-term radiological and clinical results of using the Spherus talar screw for arthroereisis, and observed improvements in radiological parameters and good clinical treatment outcomes [22]. There have been no earlier studies assessing foot function, ankle range of motion, pain severity, and physical activity levels in patients after arthroereisis with a talar screw. 

In our previous study we assessed short-term and medium-term radiological and clinical arthroereisis outcomes achieved with a talar Spherus screw in the same group of patients included in the current study [22]. We reported radiological improvement and good clinical outcomes [22].

Martinelli et al. assessed the level of sport activities in 49 patients following arthroereisis [5]. Twelve months after surgery, all patients returned to physical activity; however, the authors reported no improvement in the level of sport activities following treatment in comparison with the preoperative data [5]. The patients from our study group showed improved levels of physical activity following treatment. Arthroereisis allows for immediate full weight bearing and a rapid return to previous levels of physical and sport activities [5]. An increased level of sport activity, which may improve the emotional status of patients after treatment, is one of the goals of surgery [4,5]. To date, there have been no studies aiming to assess the levels of physical and sport activity with the UCLA activity scale, VAS, and Grimby sport activity scale after arthroereisis. Our group of patients showed significant postoperative improvement in the level of physical activity in terms of both VAS and UCLA activity scale scores, which indicated good treatment outcomes. An increase in physical and sport activity after surgery in our study population may be due to reduced pain and decreased deformity [5,13].

The main purpose of arthroereisis is to reduce pain [5,7,8,10,12,13]. Memeo reported postoperative pain in 15.8% of patients after arthroereisis with the use of a calcaneal screw, and in 5.5% of patients after arthroereisis with the use of a sinus tarsi spacer, and the mean VAS pain score was 7.4–7.8 [12]. In our study, we observed a significant postoperative reduction in pain, which is a good outcome. Pain after arthroereisis may be associated with selection of an inappropriate implant size [4,12]. 

Vogt et al. assessed 73 patients after various arthroereisis procedures with the use of various implant types [1]. Those authors reported improvement in the FFI from a mean of 36.4 prior to surgery to 22.8 after surgery [1]. In our study, we also observed a significant improvement in FFI-R scores after treatment, which may indicate that arthroereisis with the use of a talar screw yields improvement in foot function. 

Our study group showed no significant differences between the operated and non-operated limb in mean degrees of postoperative dorsiflexion, plantar flexion, adduction, or abduction at the ankle joint. Nor were there significant differences between the preoperative and postoperative ranges of motion at the ankle joint. The exception was the pre-operatively larger range of dorsiflexion in the unoperated limb (36.25°) compared to the operated limb (22°), *p* = 0.047. All joint range of motion measurements before and after surgery were performed in the same way by the same investigators. These results indicate good ranges of motion after treatment with a talar screw. Unlike wedge osteotomy and arthrodesis procedures, arthroereisis is a reversible treatment method that helps keep some range of motion at the subtalar joint [1]. On the other hand, sinus tarsi implants may cause fibular muscle contractures in 3–10% of patients [1]. In addition, Retana reported limited movement at the subtalar joint following the use of a calcaneal screw [13]. In our group of patients, we observed no cases of fibular muscle contracture, which may have been responsible for the lack of range of motion limitations at the ankle joint following treatment. The use of the Spherus screw facilitates early rehabilitation and bearing full weight on the treated limb, which may help improve the range of motion. Some of the other authors use cast immobilization for six weeks after surgery [13,14], which may also limit the ankle’s range of motion. 

Thirty-seven percent of our study group underwent simultaneous Achilles tendon lengthening. Other authors also reported performing additional procedures in 17–100% of patients undergoing arthroereisis [1,3,7,9,10,11,12,13,14,16,17,18]. Those additional procedures had no effect on either clinical or radiological outcomes [10,14]. 

In our group, none of the patients had the screw removed.

The use of a talar screw allows for other foot or ankle surgeries to be performed in the future. Tendon lengthening or transfer, osteotomy, or arthrodesis procedures may be performed without limitations.

The limitations of our study are the relatively short follow-up period and a relatively small sample size. Both are due to a prospective nature of the study, limited number of patients with indications for arthroereisis, our eagerness to present an evaluation of the novel type of implants, and the limited capacity for orthopedic surgery procedures at our hospital. However, other authors also reported a similar follow-up period [6,14,19]. On the other hand, according to Hagen, the return of foot function occurs within four weeks after arthroereisis [19]. Some authors analyzed the results based on study populations of similar size [6,9,14,16,19]. Another limitation of our study is the lack of a control group, which is the result of the prospective nature of this study; however, other authors also conducted studies without a control group [1,3,5,6,12,13,14,15,16,17,18,19]. 

The strengths of our study include its prospective nature, identical rehabilitation protocol, and the surgeries being conducted by one of only two orthopedic surgeons. Some studies on arthroereisis are retrospective [1,3,4,5,6,7,12,13,14,15,18]. In the future, we are planning a similar study in a larger group of patients and with a longer follow-up period. 

## 5. Conclusions

The use of a talar screw in the treatment of symptomatic pes planovalgus helps reduce pain and improve functional outcomes after treatment. 

Foot function assessments showed diminished pain, improved levels of physical and sport activity, and no effect on the range of motion after surgery in comparison with preoperative data. 

Arthroereisis with a talar screw is a valid surgical technique for the treatment of symptomatic pes planovalgus.

## Figures and Tables

**Table 1:** Detailed functional assessment of patients before and after surgery.

Analyzed Variable	Before Treatment	After Treatment	*p* Value *
	(Mean ± Standard Deviation)		
UCLA scale	4.78 ± 2.25	6.05 ± 1.61	0.045
Gimbry scale	3.26 ± 1.59	3.94 ± 1.17	0.14
VAS Activity Scale	5.47 ± 2.89	7 ± 2.05	0.048
VAS pain scale	4.73 ± 3.51	2.73 ± 3.28	0.047
FFI-R scale	140 ± 61.28	97.75 ± 28.53	0.017
Dorsiflexion OL [degree]	22 ± 8.44	25.33 ± 6.77	0.316
Plantar flexion OL [degree]	30.7 ± 9.21	32.91 ± 11.22	0.623
adduction OL [degree]	21.9 ± 9.74	23.5 ± 7.46	0.667
abduction OL [degree]	18.6 ± 9.92	17.83 ± 6.72	0.831
Dorsiflexion NOL [degree]	36.25 ± 8.53	29 ± 13.41	0.381
Plantar flexion NOL [degree]	37 ± 14.58	49 ± 19.49	0.342
adduction NOL [degree]	28.5 ± 6.24	25 ± 3.53	0.32
abduction NOL [degree]	28.5 ± 12.61	22 ± 10.36	0.422

* U Mann–Whitney Test; OL—operated limb, NOL—non-operated limb.

**Table 2:** Detailed range of motion of patients before and after treatment.

Analyzed Variable	(Mean ± Standard Deviation)		*p* Value
before treatment			
	OL	NOL	
Dorsiflexion [degree]	22 ± 8.44	36.25 ± 8.53	0.047
plantar flexion [degree]	30.7 ± 9.21	37 ± 14.58	0.343
abduction [degree]	18.6 ± 9.92	28.5 ± 12.61	0.142
adduction [degree]	21.9 ± 9.74	28.5 ± 6.24	0.238
after treatment			
	OL	NOL	
Dorsiflexion [degree]	25.33 ± 6.77	29 ± 13.41	0.457
plantar flexion [degree]	32.91 ± 11.22	49 ± 19.49	0.086
abduction [degree]	17.83 ± 6.72	22 ± 10.36	0.335
adduction [degree]	23.5 ± 7.46	25 ± 3.53	0.677

OL—operated limb, NOL—non-operated limb.

## Data Availability

The data presented in this study are available on request from the corresponding author.
